# Oncogenic-tsRNA: A novel diagnostic and therapeutic molecule for cancer clinic

**DOI:** 10.7150/jca.98656

**Published:** 2024-08-19

**Authors:** Lin Chen, Yao Wu, Qi Tang, Faqing Tang

**Affiliations:** 1 The First Clinical College of Hunan University of Chinese Medicine & Hunan Cancer Hospital, Changsha, 410007, China.; 2 Hunan Key Laboratory of Oncotarget Gene and Clinical Laboratory of the Affiliated Cancer Hospital of Xiangya School of Medicine, Central South University, Changsha 410013, China.

**Keywords:** tsRNAs, tumor, diagnosis, targeted therapy, biomarker

## Abstract

tsRNA (tRNA-derived small RNA) is derived from mature tRNA or precursor tRNA (pre-tRNAs). It is lately found that tsRNA's aberrant expression is associated with tumor occurrence and development, it may be used a molecule of diagnosis and therapy. Based on the cleavage position of pre-tRNAs or mature tRNAs, tsRNAs are classified into two categories: tRNA-derived fragments (tRFs) and tRNA halves (also named tiRNAs or tRHs). tsRNAs display more stability within cells, tissues, and peripheral blood than other small non-coding RNAs (sncRNAs), and play a role of stable entities that function in various biological contexts, thus, they may serve as functional molecules in human disease. Recently, tsRNAs have been found in a large number of tumors including such as lung cancer, breast cancer, gastric cancer, colorectal cancer, liver cancer, and prostate cancer. Although the biological function of tsRNAs is still poorly understood, increasing evidences have indicated that tsRNAs have a great significance and potential in early tumor screening and diagnosis, therapeutic targets and application, and prognosis. In the present review, we mainly describe tsRNAs in tumors and their potential clinical value in early screening and diagnosis, therapeutic targets and application, and prognosis, it provides theoretical support and guidance for further revealing the therapeutic potential of tsRNAs in tumor.

## Introduction

tRNA-derived small RNAs (tsRNAs) are a novel class of functional RNA molecules that are derived from mature tRNAs or precursor tRNAs (pre-tRNAs), and are aberrantly expressed under various conditions, such as ultraviolet radiation, arsenite, heat shock, hypoxia, oxidative damage or viral infection 1-4. tRNAs are initially transcribed by RNA polymerase III in the nucleus in the form of pre-tRNAs 5. Mature tRNAs are usually 73-93 nucleotides (nts) in length and are characterized by a cloverleaf-shaped secondary structure with five arms designated as amino acid, including D, anticodon, variable and the TψC arms, respectively. Any additional nucleotides beyond nucleotide 73 are accommodated in the variable-loop or in the D-loop. Two of the tRNA arms are critical for its functions as an adaptor: the amino acid arm carrying a specific amino acid and the anticodon arm containing an anticodon that recognizes the codon in template mRNA. Moreover, the cloverleaf secondary structure is folded into an L-shaped tertiary structure with an amino acid arm and an anticodon arm located at separate ends 5. With rapid advances in high-throughput sequencing technologies, many studies have reported that tsRNAs participate in the cell biology including gene regulation, transposon repression, and disease onset and progression 6-9. Since tsRNAs are derived from tRNAs with heavy modifications and characteristic structures, they display more stability in cells, tissues, and peripheral blood than other small non-coding RNAs (sncRNAs) 10. The heterogeneous population of tsRNAs represents distinct and stable entities that function in various biological contexts, including stress responses, tumorigenesis, stem cell biology, and epigenetic inheritance 11. Thus, tsRNA may serve as marker molecules in human disease. Accumulating evidence has demonstrated that tsRNAs play a critical role in human cancer 3, 4, 12-19, with its potential as a biomarker for early screening, diagnosis and prognosis of tumors, but also as an important target for tumor therapy.

tsRNA belongs to one species of sncRNA. sncRNAs include microRNA (miRNA), small interfering RNA (siRNA), small nucleolar RNA (snoRNA), small nuclear RNA (snRNA), PIWI-interacting RNA (piRNA), and RNA-derived small RNA (tsRNA). Short or small interfering RNAs (siRNAs) and microRNAs (miRNAs) are molecules similar in size 20. The expression of some snoRNAs is cell type specific and their biogenesis is dynamically regulated during body development. snoRNA is stable, which occurs mainly by binding with proteins to form a complex that enhances stability, however, the specific mechanism of the binding remains unclear 21. The biogenesis of snRNA is regulated by specific developmental phenomenas (such as being regulated by certain cellular stress conditions, or closely related to RNA polymerase II) and is largely conserved in its expression 21. The piRNA has permanently been considered germline-specific. With further studies, the tissue specificity of somatic piRNA has been confirmed. Mature piRNA stability is mainly achieved by methylation methyltransferase 2'-o-methylated, and while the potential mechanisms of the biogenesis (such as ethylation modification or other modifications) have been less studied 21. The tsRNAs have different functions from the other sncRNAs (siRNA, miRNA, snoRNA, snRNA, and piRNA). However, tsRNAs regulate gene expression mainly at post-transcription suppression, post-transcriptional, translation and post-translational, and reverse transcription regulation. siRNAs and miRNAs are complementary to its corresponding mRNA sequences, which can inhibit gene expression, inducing the degradation of the transcript or the inhibition of their translation. siRNAs bind specifcally to a single gene location by sequence complementarity and regulate gene expression by specifcally targeting transcription units to silence osttranscriptional gene. miRNAs can regulate the expression of diferent gene targets through their imperfect base pairing. siRNAs and miRNAs have been extensively studied in the past decades and their contribution to the development of various pathologenses are now well established for the treatment of different diseases including cancer 20. snoRNAs guide post-transcriptional covalent modifications that alter RNA biophysical properties and can also modify each other's modification. In addition, selective splicing can also be regulated 21. piRNAs can interact with PIWI protein to silence transposons. After translation, piRNAs can regulate the stability of its interacting proteins by binding to proteins 21. Some tsRNAs can also act as a piRNA to interact with PIWI protein to regulate gene expression at the transcriptional level. tsRNA can interact with Argonaute protein to form RNA-induced silencing complex and inhibit gene expression through RNA interference. Besides, tsRNA can be used as a protein bait to affect the stability of mRNA. tsRNA can inhibit translation initiation either through binding to translation initiation complex or inhibiting transcription initiation. During retroviral cycle, cellular tRNAs serve as primers for reverse transcription in the synthesis of synthesise minus-strand cDNA, and are placed onto the site of the viral RNA. These indicate that tsRNA plays a double-edged sword in reverse transcription regulation. In the present review, we mainly describe various tsRNA in tumor and their function mechanism in tumorigenesis. The potential clinical significance of tsRNAs were evaluated in cancer early-screening and diagnosis, targeted therapy and application, and prognosis. This article provides a theoretical support and guidance for further revealing the clinical potential of tsRNAs in tumors.

## Classification and biogenesis of tsRNA

**Figure 1:**
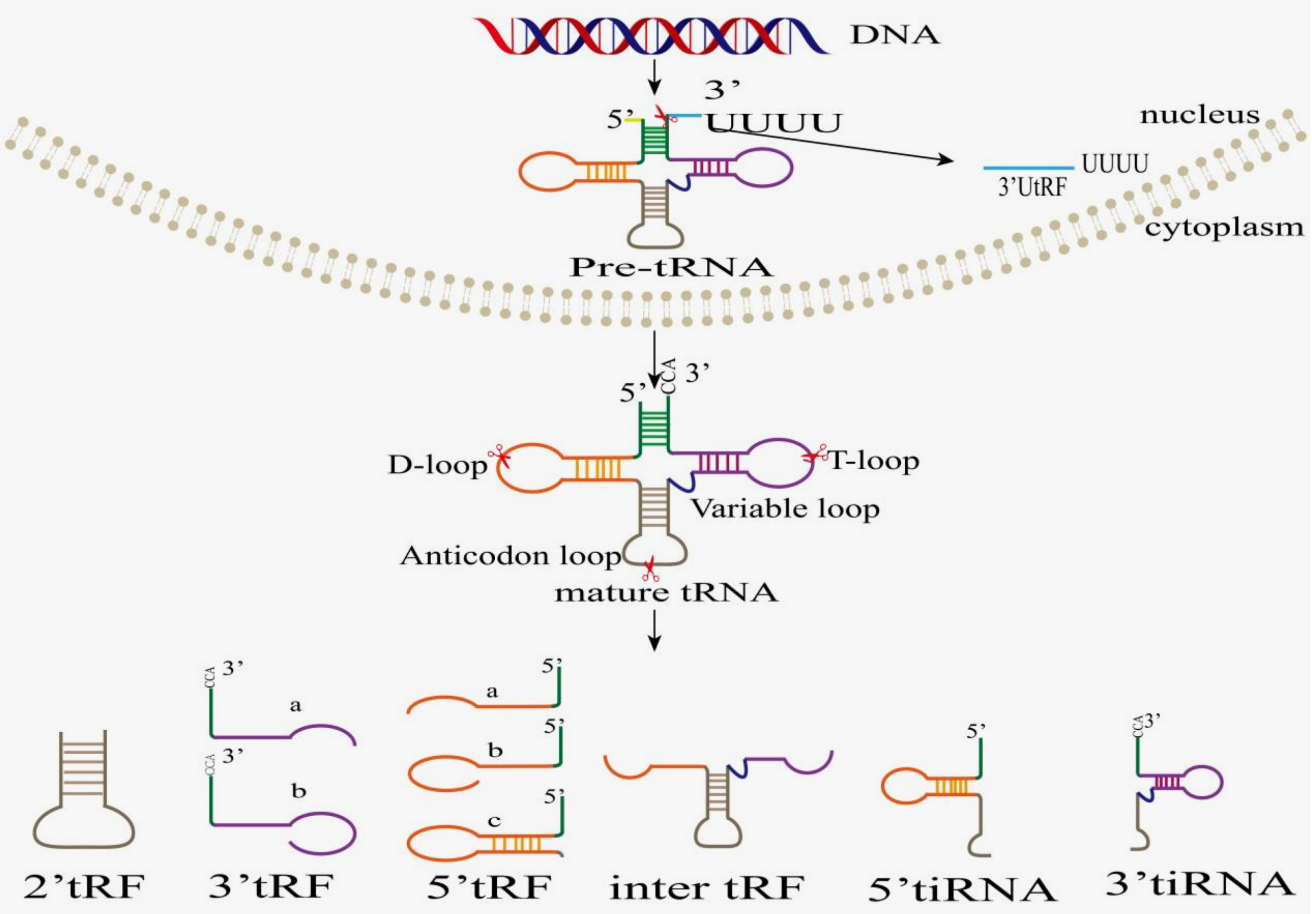
Classification and biogenesis of tsRNAs. After being transcribed by RNA polymerase III in nucleus, pre-tRNA undergoes 5'leader, 3'tailer, and intron sequence removing, as well as 3'CCA plus and modification before tRNA maturation. Ribonuclease cleavage in specific region of pre-/mature tRNA produces different types of tsRNA, including 3'UtRF(1-tRF), 5'Trf, 3'tRF, 2'tRF, i'tRF, 5'tRH (5'tiRNA), and 3'tRH (3'tiRNA).

It is worthwhile to note that tsRNAs are neither remnants of tRNA maturation, nor are they random tRNA degradation products. Conversely, tsRNAs are generated by cleavage of pre-tRNAs or mature tRNAs at specific sites, and their appearance is not accompanied by a significant depletion of their cognate tRNAs 22, 23. Based on the cleavage position of pre-tRNAs or mature tRNAs, tsRNAs are classified into two categories, tRNA-derived fragments (tRFs) and tRNA halves (also named tiRNAs or tRHs) (Figure 1). Based on their mapping positions on pre-tRNAs and mature tRNAs, tRFs and tiRNAs are divided into several subclasses 24. tRFs are non-coding RNAs with approximately 14-30 nt in length, and they are grouped into 5 subclasses: tRF-1s (1'tRFs), tRF-3s (3'tRFs), tRF-5s (5'tRFs), tRF-2s (2'tRFs), and i-tRFs (inter tRFs, i'tRFs)^25^. tRF-1s (also named 3′U-tRFs) are derived from the 3'-end of pre-tRNA mainly through endonuclease Z (RNase Z/ELAC2) digestion 24, 26-30, which results in the presence of poly-U residues at the 3'end of tRF-1s 31. tRF-2s only contain anticodon stem-loop sequences and are induced in hypoxic conditions 3. tRF-3s come from TψC-loop cleavage of mature tRNAs by Dicer or other unknown nucleases 32-34, and they are classified into two subtypes, tRF-3a and tRF-3b, with lengths of 18 and 22 nucleotides, respectively. The 18-nucleotide tRF-3a is generated by the cleavage between 58th and 59th nucleotides on the TΨC-loop. The 22-nucleotide tRF-3b is produced by cleavage between 54th and 55th nucleotides on the TΨC-loop. The end of TΨC-loop sequence contains CCA sequence, which is added to TΨC-loop during mature tRNA processing. tRF-5s minimally extend to D-loop and maximally to anticodon stem, and they are classified into tRF-5a (14-16 nt), tRF-5b (22-24 nt), and tRF-5c (28-30 nt) subtypes 35 based on the cleavage by Dicer, RNase T2, or RNase A, respectively 32, 33, 35. The i-tRFs originate from the internal body of mature tRNAs, straddling anticodon region due to cleavage by unknown nucleases 26 and including anticodon-loop and part of D/T-loop. Its production may be related to hypoxia stress stimulation, but the specific mechanism remains unclear. tiRNAs are non-coding small RNAs with 31-40 nucleotides in length, which can be classified into 3'-tiRNAs and 5'-tiRNAs 24, 26-30. As the earliest discovered tsRNAs, tiRNAs are produced by cleavage specifically in the anticodon-loop of mature tRNAs 36**.** Despite being known as stress-induced fragments, tiRNAs also exist in non-stressed conditions 37. 5'tiRNA replication starts from the 5'end of mature tRNAs and end at the anticodon-loop. Similarly, 3'tiRNA replication starts from the anticodon-loop and end at the 3'end of mature tRNAs. 5'tRNA halves (31-40 nt) are produced by specific cleavage within the anticodon-loop of mature tRNA under various stress conditions, such as heat shock, hypoxia, UV irradiation, oxidative stress, amino acid/glucose starvation, and viral infection. Therefore, these tsRNAs are also called tiRNA-5s. 3'tRNA halves (31-40 nt), also known as tiRNA-3s, are generated by specific cleavage within anticodon-loop during stress responses 22, 38-41. Angiogenin (ANG), RNase T2, and RNase L are responsible for tiRNA biogenesis 22, 37-43. The biogenesis of tsRNA is enzymatically modified: the biogenesis of tsRNA is affected by modifying enzymes, but the specific mechanisms by which these modification enzymes recognize tRNA targets and induce modification to produce the corresponding tsRNA remain unclear.

## tsRNAs in tumors

tsRNA belongs to a species of small non-coding RNA, a derivative of pre-tRNA or mature tRNA. Some studies have found that tsRNA is abnormally expressed in many tumor tissues, cells, or peripheral blood (Table 1).

## tsRNAs potentially used for tumor early-state screening and diagnosis

**Figure 2:**
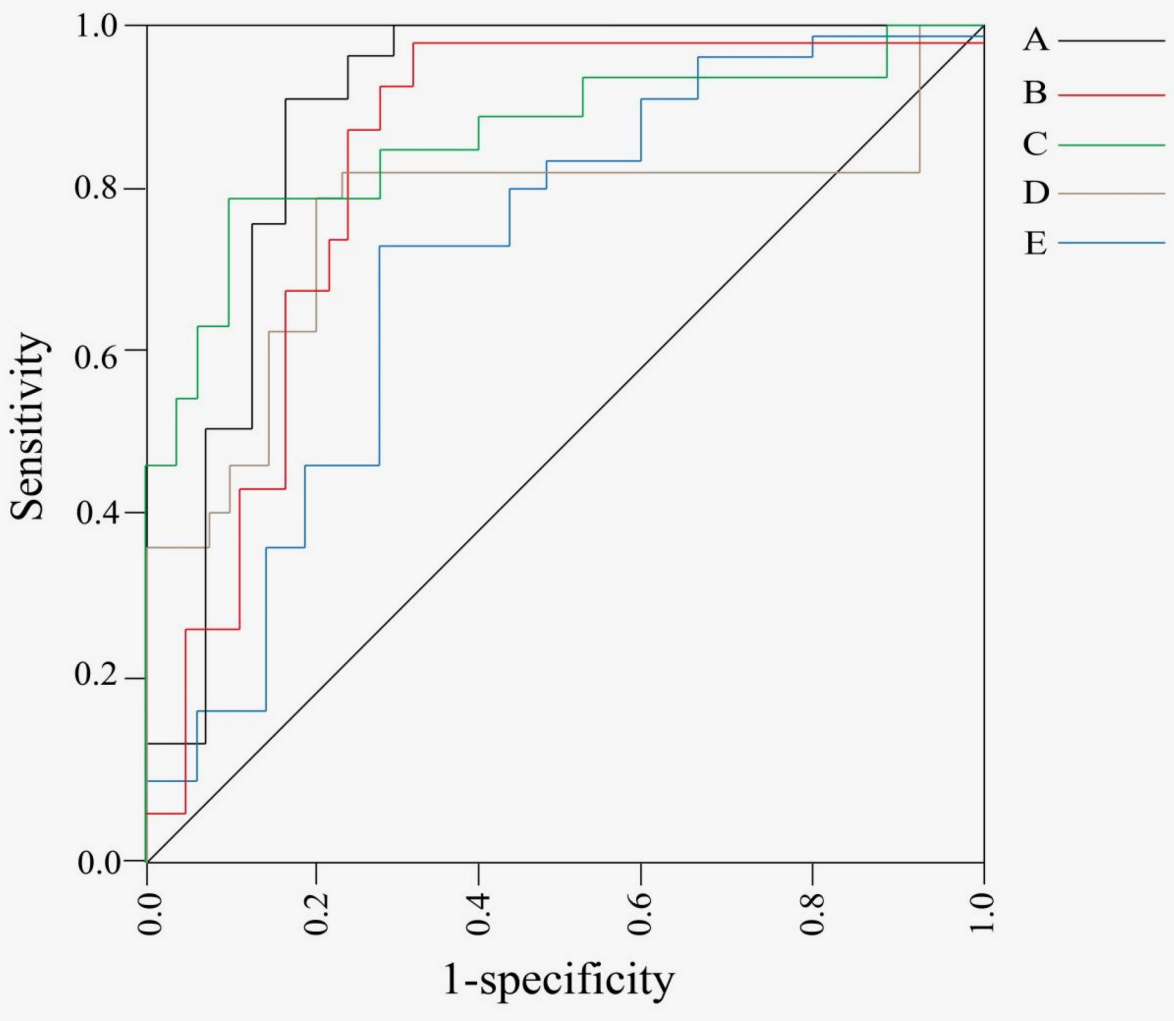
ROC curve of tsRNAs of tumor early-stage screening and diagnosis.** A,** According to ROC curve analysis, tRF-22-WB86Q3P92, tRF-22-WE8SPOX52, tRF-22-WE8S68L52, and tRF-18-8R1546D2 may have some diagnostic and prognostic potential in CRC *(Reproduced with permission from Front Oncol publisher, the link of the Creative Commons licence:* http://creativecommons.org/licenses/by/4.0/.) 63. **B,** According to ROC curve analysis, tRNA-GlyGCC-5 has a value as a preoperative biomarker for ESCC diagnosis *(Reproduced with permission from Mol Cancer publisher, the link of the Creative Commons licence:* http://creativecommons.org/licenses/by/4.0/.) 85.** C,** According to ROC curve analysis, tRFPro-AGG-004 and tRF-Leu-CAG-002 has useful as a potential diagnostic biomarker for PC *(Reproduced with permission from Mol Cancer publisher, the link of the Creative Commons licence:* http://creativecommons.org/licenses/by/4.0/.) 68. **D,** According to the ROC curve, tRF-1:28-Val-CAC-2 may be a potential diagnostic marker in NPC cases *(Reproduced with permission from Front Mol Biosci publisher, the link of the Creative Commons licence:* http://creativecommons.org/licenses/by/4.0/.) 81. **E,** According to ROC curve, tRF-Arg-CCT-017, tRF-Gly-CCC-001 and tiRNA-Phe-GAA-003 can be used as a diagnostic index for BC *(Reproduced with permission from NPJ Breast Cancer publisher, the link of the Creative Commons licence:* http://creativecommons.org/licenses/by/4.0/.) 49.

In the clinic, a large number of tumors are discovered and diagnosed at advanced stage, and have already missed the best opportunity for treatment. Therefore, improving the early screening rate and diagnosis rate has become the focus of current cancer treatment research. Recently, the discovery of tsRNA and its abnormal expression in tumors can effectively distinguish tumor patients from healthy people. And tsRNA may be a new diagnostic biomarker for tumors, and may be used for tumor early-state screening in theory. Many studies have found a significant rise of tsRNA expression in tumor tissue, cell line, or serum (Table 2). Such as, the expression of tRF-Leu-CAG was significantly upregulated in NSCLC tissues, cell lines and serum and was positively correlated with tumor stage, which proved that tRF-Leu-CAG may be used as a diagnostic marker in stage NSCLC IV cases 44. Meanwhile, some studies found that tRF-21-RK9P4P9L0 and tRF-16-PSQP4PE were highly elevated in LUAD tissues compared with normal tissues. These findings indicated that tsRNA can be utilized as a diagnostic biomarker for LUAD, but a single tsRNA is less efficient than a combination of multiple tsRNAs 46. The expression levels of tRF-Arg-CCT-017 in HER-2 subtypes, and expression levels of tRF-Gly-CCC-001 and tiRNA-Phe-GAA-003 differ between luminal BC and TNBC, reflecting obvious heterogeneity, suggesting that tRF-Arg-CCT-017, tRF-Gly-CCC-001 and tiRNA-Phe-GAA-003 can serve as novel diagnostic biomarkers for BC (Figure 2E) 49. Moreover, tRF-1:28-Val-CAC-2 has a relatively good ability to distinguish between primary NPC and healthy control (Figure 2D) 81. tRF-39-0VL8K87SIRMM12E2 was verified by qPCR to be significantly upregulated in PTC cell lines and tissue samples 83. In the tsRNAs used in diagnosis, ts-N102 was significantly upregulated in HCC tissues, while the highly related hsa-mir-215 was a tumor suppressor in CRC and multiple myeloma 60. As an important part of the biobank, blood sample has rich biomolecules that are used for disease diagnosis, stage identification, and prognosis prediction.

Compared with tissue sample, blood sample has advantages of easy access, continuous sampling, and high patient acceptance. Therefore, simultaneous analysis of biomarkers found that tsRNAs in blood sample may improve an accuracy and convenience of cancer detection (Table 2). Some reports suggested that half of 5′-tRNA-Arg-CCT, 5'-tRNA-Glu-CTC, and 5'-tRNA-Lys-TTT were downregulated in clear cell RCC patient serum and tissues, possibly as a non-invasive biomarker 69. RNAs in exosomes are relatively more stable, more resistant to physical degradation, and exosomes are easily accessible and carry a variety of molecules associated with specific diseases. The four tsRNA (tRNA-ValTAC-3, tRNA-GlyTCC-5, tRNA-ValAAC-5, and tRNA-GluCTC-5) were identified to be highly expressed in exosomes derived from the plasma of HCC patients 62. The saliva-derived exosomal tsRNA (tRNA-GlyGCC-5) was found to be could distinguish in the patients with ESCC, and it was served as a non-invasive, convenient and reliable diagnostic biomarker. tRNA-GlyGCC-5 was supposed to be a preoperative biomarker to select patients who benefit from adjuvant therapy (Figure 2B) 85. In EC patients, the exosomal tRF-20-S998LO9D could potentially be used as a non-invasive biomarker 72.

tsRNA is a novel type of regulatory non-coding RNA that has attracted a great attention across multiple subfields of biology, particular in recent studies to its participation in a variety of biological processes under diverse pathological and physiological conditions (Table 2). Yue Huang *et al.* used bioinformatics analysis to predict that tDR-7816-mediated xenobiotic metabolic processes supporting tumorigenesis in BC, and identified tDR-7816 as a potential biomarker and intervention target for non-TNBC 51. tsRNA-26576 may act as an oncoprotein by inhibiting the expression of SPEN and FAT4. Normally, tsRNA remains stable and detectable in the blood, and its dysregulation has been identified to be associated with BC development or progression, suggesting that tsRNA-26576 may be a valid marker for BC diagnosis 47. In OSF formation, tRF-Gly-TCC-016 may promote OSF formation and progression through cytokine-cytokine receptor interaction and cAMP signaling pathway, so it was documented to have an important significance in early screening and diagnosis of OSF, and may serve as a potential diagnostic marker 82. In addition, androgen-dependent tsRNAs (5'-tRNA-Glu-CUC) may be used as a biomarker to monitor and predict progression in PC 70.

Moreover, tsRNA expression is tissue specific, particularly spatiotemporal, and many studies have emerged on tsRNA as a clinical marker (Table 2). Some studies found that combining 2-tsRNAs features have some disease specificity in PC, suggesting that serum tRFPro-AGG-004 and tRF-Leu-CAG-002 can serve as a new promising biomarker, even in early-stage screening (Figure 2C) 68. In addition, some specific tsRNAs (tsRNA-ValTAC-41 and tsRNA-MetCAT-37) may be highly sensitive, non-invasive, and effective biomarkers for PDAC 67. Bing Xu *et al.* identified four tRNA fragments from tRNA-Leu-CAA in sncRNA-seq dataset of glioma samples, and found that three tsRNAs (ts-26, tRFdb-3012a and tRFdb-3012b) were significantly downregulated in glioma, indicating that tRNA-leu-caa-derived tsRNA may serve as a diagnostic and prognostic biomarke for diffuse glioma 76. Hongxia Deng *et al.* identified a 5′-tiRNA, tRF-33-Q1Q89P9L842205, which is specifically cleaved by angiogenin in the anticodon of mature tRNA-Gly-CC and closely associated with LSCC, while tRF-33-Q1Q89P9L842205 has promising applications as a biomarker of LSCC 84. tRF-Gln-TTG-006 is significantly better diagnostic than AFP in the early stage of HCC 61. tRF-22-WB86Q3P92, tRF-22-WE8SPOX52, tRF-22-WE8S68L52, and tRF-18-8R1546D2 were found to have some diagnostic and prognostic potential for in CRC (Figure 2A) 63.

## tsRNA-targeted therapy in tumors

**Figure 3:**
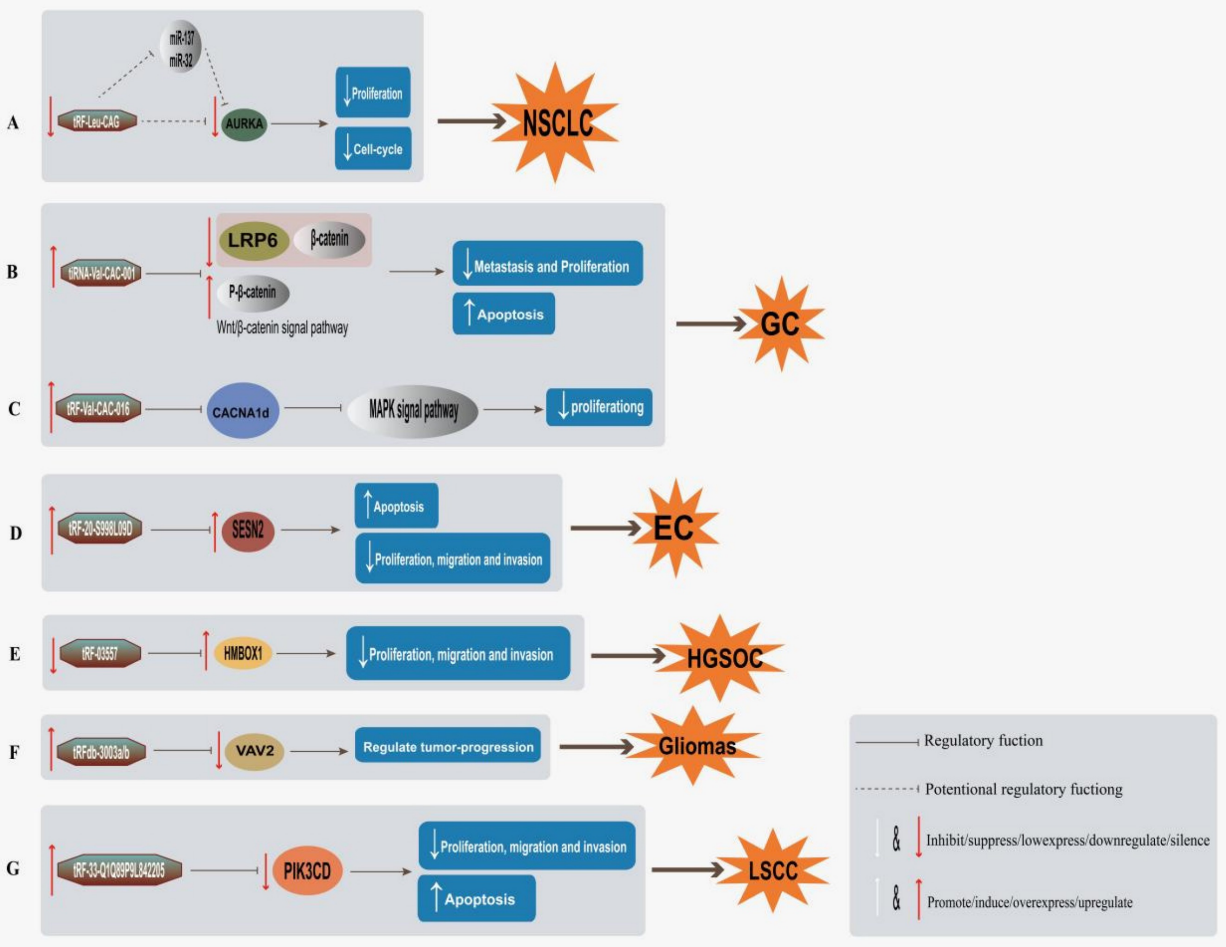
tsRNAs used for tumor targeted therapy and mechanism. **A,** Inhibition of tRF-Leu-CAG expression in NSCLC can inhibit cell proliferation and hinder cell cycle by direct downregulation of AURKA expression or inhibition of AURKA expression by regulating other genes involved in cancer process 44; **B,** tiRNA-Val-CAC-001 downregulates protein level of LRP 6 and β-catenin, while upregulates p-β-catenin, inhibiting cell migration and proliferation and promoting apoptosis of GC cells 53; **C,** tRF-Val-CAC-016 regulates MAPK pathway by targeting CACNA1d, thereby inhibiting GC cell proliferation 54; **D,** Overexpression of tRF-20-S998LO9D upregulates SESN2 expression, thus inhibiting the proliferation, migration and invasion of EC cells, and promoting cell apoptosis 72; **(E)** Inhibition of tRF-03357 expression directly downregulate HMBOX1 in HGSOC to inhibit cell proliferation, migration and invasion 73; **F,** Overexpression of tRFdb-3003a/b directly binds to VAV 2 and downregulates VAV 2 expression in gliomas, thus regulating tumor progression 74; **(G)** tRF-33-Q1Q89P9L842205 inhibits cell growth, proliferation, migration, invasion and induced apoptosis of LSCC cells by directly silencing PIK3CD 84.

Some tsRNAs have been described as functional molecules to promote cancer progression, rendering them as promising therapeutic targets or agents (Table 3). For example, in NSCLC, tRF-Leu-CAG could suppress the proliferation of NSCLC cells and inhibit G0/G1 cell-cycle progression through targeting AURKA (Figure 3A) 44. The 5'tiRNA-His-GTG can inhibit the expressions of pro-proliferation and anti-apoptosis related genes through hippo signaling pathway by targeting LATS2 66. tRF-03357 has predictive targeting, while inhibition of tRF-03357 expression can directly upregulate HMBOX1 in HGSOC to inhibit cell proliferation, migration and invasion (Figure 3E) 73. These studies indicate that tsRNAs have great potential as therapeutic drugs. However, there is still a long way to go before clinical transformation application.

Some tsRNAs can not only promote cell proliferation as a carcinogen, but also inhibit the development of cancer as an inhibitor. tsRNA could realize its targets through regulating a certain target gene protein by some tumor signaling pathways, and then achieve an targeted therapeutic effect (Table 3). For example, tiRNA-Val-CAC-001 works as a cancer suppressor in GC by targeting LRP6 via Wnt/β-catenin signaling pathway, and upregulating tiRNA-Val-CAC-001 inhibited metastasis and proliferation but promoting apoptosis (Figure 3B) 53. Meanwhile, tRF-Val-CAC-016 in GC was found to modulate CACNA1d-mediated transduction of MAPK signaling pathway, thus inhibiting the proliferation of GC (Figure 3C) 54. tsRNA may also directly upregulate or downregulate the related target genes to inhibit tumor progression (Table 3). For example, tRF-20-S998LO9D is an EC repressor, inhibits migration, proliferation and invasion, and promotes apoptosis by upregulating SESN2 (Figure 3D) 72. The tRF-33-Q1Q89P9L842205 induces apoptosis in LSCC cells and inhibits cell growth, migration and invasion via directly downregulating PIK3CD expression in LSCC (Figure 3G) 84. Furthermore, Jian Ren *et al.* found that overexpression of tRFdb-3003a/b may play a key role in tumor progression of gliomas, tRFdb-3003a/b may inhibit tumor proliferation and growth by directly binding to VAV2 to regulate VAV2 expression in gliomas (Figure 3F) 74. Meanwhile some investigators have also found that downregulated tRFdb-3012a/b may directly target the 3'untranslated region of RBM 43, and ts-26 may directly target the 3'untranslated region of HOXA13 to play a tumor suppressor role in glioma progression through specific signaling pathways 76. Clinically, there are also some specific targets and pathways of tsRNA that have not yet been discovered, but they play an important role in cancer therapy through some specific mechanisms of action (Table 3). For example, in the transformation of OSF to oral squamous cell carcinoma (OSCC), tiRNA-Val-CAC-002 acts as a suppressor of oncogenic cytokines, which affects the course of OSCC by regulating tumor cell proliferation and invasion and mesenchymal-epithelial transformation (MET) 82. Some reports found that ts-46 and ts-47 strongly downregulated in invasive CLL 78, suggesting that these tsRNAs are potential tumor suppressors, meanwhile, ts-42 was inactivated in CLL mainly through promoter methylation, thus acting as tumor suppression 79. tRF-0009 (tDR-7336) may play a key regulatory role in hypoxia-induced chemoresistance in TNBC 50. These studies suggest that, in tumor therapeutic strategy, tsRNAs may inhibit tumor onset or progress mainly through suppressing tumor cell proliferation, growth, migration, and invasion, and promoting cell apoptosis, to achieve therapeutic effects.

## tsRNAs used for prognosis of the patients with tumor

**Figure 4:**
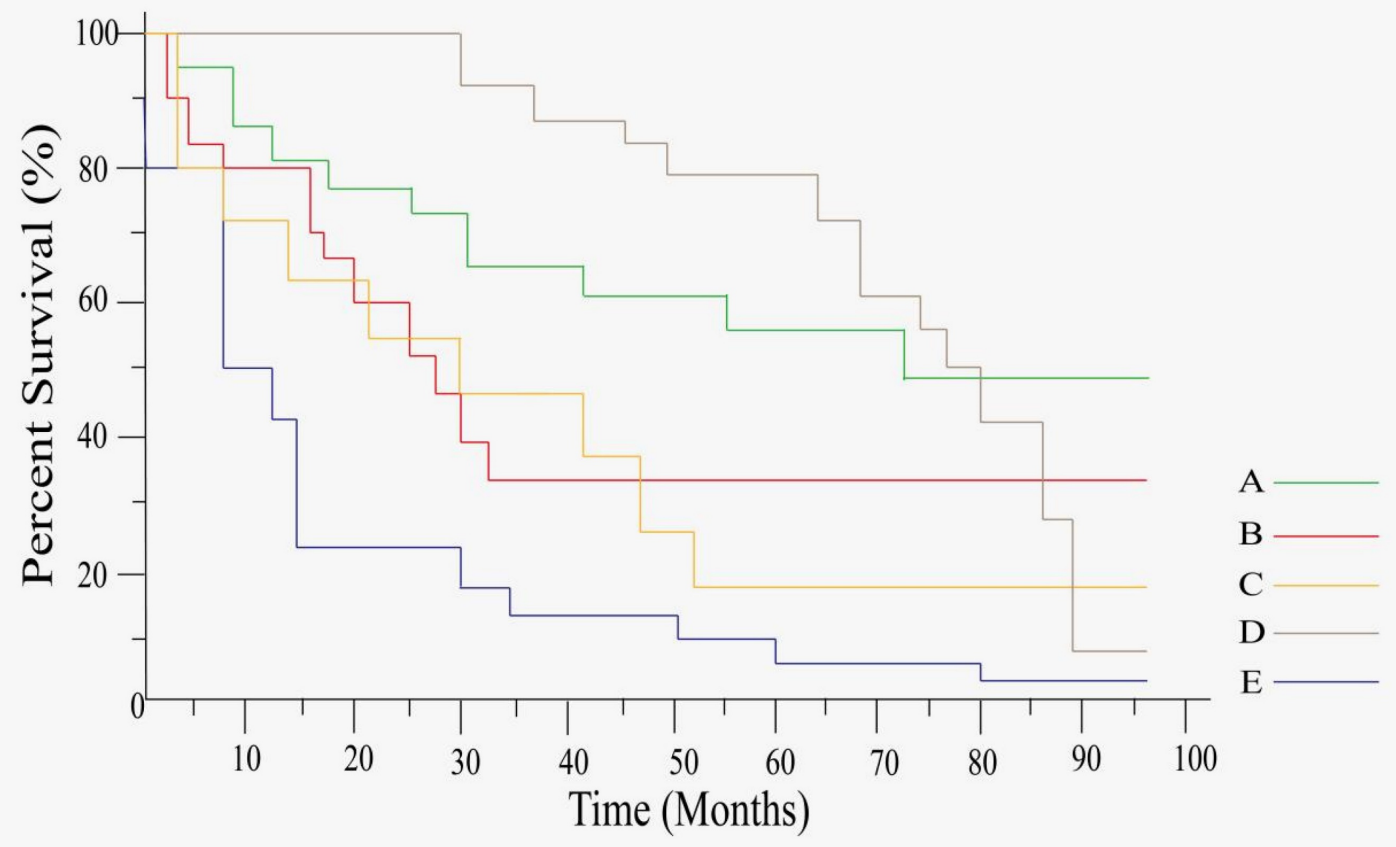
Kaplan-Meier curve analysis of tsRNAs expression for OS. **A,** 5'tiRNA-Pro-TGG is negatively associated with CRC prognosis *(Reproduced with permission from Cancer Cell Int publisher, the link of the Creative Commons licence:* http://creativecommons.org/licenses/by/4.0/.) 64. **B,** tRF-Pro-AGG-004 and tRF-Leu-CAG-002 are negatively associated with PC prognosis *(Reproduced with permission from Mol Cancer publisher, the link of the Creative Commons licence:* http://creativecommons.org/licenses/by/4.0/.) 68. **C,** ts-26 and tRFdb-3012a/b are positively associated with Diffuse glioma prognosis *(Reproduced with permission from Cancer Manag Res publisher, the link of the Creative Commons licence:* https://www.dovepress.com/terms.php & http://creativecommons.org/licenses/by-nc/3.0/.) 76. **D,** tRF-33-6SXMSL73VL4YDN, tRF-32-6SXMSL73VL4YK, tRF-32-M1M3WD8S746D2, tRF-35-RPM830MMUKLY5Z, tRF-33-K768WP9N1EWJDW, and tRF-32-MIF91SS2P46I3 are negatively associated with Osteosarcoma prognosis *(Reproduced with permission from Front Oncol publisher, the link of the Creative Commons licence:* http://creativecommons.org/licenses/by/4.0/.) 77. **E,** tRF-21-RK9P4P9L0 is negatively associated with LUAD prognosis *(Reproduced with permission from Cancer Cell Int publisher, the link of the Creative Commons licence:* http://creativecommons.org/licenses/by/4.0/.) 46.

Increasing number of reports indicate that tsRNA has a great potential as biomarker for tumor prognosis. Some tsRNAs are downregulated in tumors, they are associated with higher survival in cancer (Table 4). Zuo *et al.*, using multivariate COX survival analysis, raised that one tsRNAs (ts-N22) was poorly expressed and was associated with a lower overall survival rate in patients with HCC 60. Xu *et al.* found that the tumor patients with three tsRNAs (ts-26, tRFdb-3012a, and tRFdb-3012b) had significantly lower clinical survival than those with high expression through Kaplan-Meier curve analysis and log-rank comparison, indicating that downregulated tsRNAs (ts-26 and tRFdb-3012a/b) were associated with poor survival prognosis in glioma patients (Figure 4C) 76.

Moreover, Bill M *et al.* found that only KD of tsRNA20 and tsRNA66 reduced the proliferation capacity of THP-1 and OCI-AML 3 cells, indicating that upregulated tsRNAs (tsRNA20 and tsRNA66) improved the overall survival of CN-AML 80. Some tsRNAs highly expressed in the tumors, and they were also associated with the lower survival of the tumor patients (Table 4). Hu *et al.* reported that tsRNA-5001a was significantly upregulated in LUAD tissues, and function assay showed that overexpression of tsRNA-5001a significantly increased the risk of postoperative recurrence in LUAD patients and was associated with poor prognosis 45. Meanwhile, Wang *et al.* showed that, in LUAD tissue, only tRF-21-RKP4P9L0 was highly elevated and significantly associated with a poor prognosis, in mechanism, overexpression of tRF-21-RK9P4P9L0 promotes the proliferation, migration and invasion of LUAD (A549 and H1299) cells (Figure 4E) 46. Wang *et al.* showed that the expression of tiRNA-1:33-Pro-TGG-1 (5′tiRNA-Pro-TGG) was higher in SSLs than that in healthy population, while 5′tiRNA-Pro-TGG and heparanase 2 (HPSE2) were found a significant negative correlation between their expression levels, another analysis of survival outcomes in CRC patients demonstrated that the lower level of HPSE2 was associated with poorer prognosis (Figure 4A) 64. Wang *et al.* found that the breast cancer patients with higher levels of tRF-Arg-CCT-017 or tiRNA-Phe-GAA-003 were associated with worse disease-free survival rate (DFS) and overall survival rate (OS) through statistical Kaplan-Meier curves to analyze DFS and OS 49. Xue *et al.* found the patients with low serum tsRNA-ValTAC-41 level had a significantly longer OS than those with high level 67. Jin *et al.* found that tRF-Pro-AGG-004 and tRF-LeuCAG-002 played a cancer-promoting role in PC, with which the patients have a poor prognosis (Figure 4B) 68. Tang *et al.* identified six high tsRNAs including tRF-33-6 SXMSL-6VL4YDN, SXMSL73VL4YK, tRF-32-M1M-MWD8S746D2, tRF-35-RPM830-MMUKLY5Z, tRF-33-K768WP9N1EWJDW, and tRF-32-MIF91SS2P46I3 in osteosarcoma, they were associated with poor survival in osteosarcoma patients (Figure 4D) 77. Furthermore, Balatti *et al.* found that ts-3676 and ts-4521 had a prognostic value for specific cell subgroups of CLL, with ts-3676 and ts-4521 almost twice the patients showing both aggressive clinical course and markers in patients with poor prognostic markers but indolent disease 78.

## Conclusions and prospects

tsRNA has now attracted significant interest, establishing diverse tsRNA types and abundance of functions. According to tsRNA corresponding positions in the parental tRNA transcripts, tsRNAs are classified into tRF-1s, tRF-3s, tRF-5s, tiRNA, and tRF-2s/i-tRFs. Each particular type of tsRNA has a specific structure and production process. tsRNA is expected to be used as a marker for early-stage screening, diagnosis and prognosis of tumors, and as a target for treatment, with broad application prospects. However, the exact underlying mechanisms of action and the corresponding functions of each tsRNA type have not been fully elucidated. Therefore, further researches are needed to elucidate tsRNA mechanism in tumorigenesis and estimate tsRNA's value in clinical application. Meanwhile, with the development of high-throughput sequencing technology and the emergence of even more advanced research methods, increasingly more tsRNA will be discovered, with a high diversity of types and functions, which would extend their potential beneficial applications.

## Figures and Tables

**Table 1:** tsRNAs in tumors

Tumor type	tsRNA	tsRNA type	Specimen	Refs
Non-small cell lung cancer (NSCLC)	tRF-Leu-CAG	tiRNAs	NSCLC tissues, cell lines, and sera	44
Lung adenocarcinoma (LUAD)	tsRNA-5001a	-	LUAD tissues	45
	tRF-16-L85J3KE	i-tRF	LUAD tissues and cell lines	46
	tRF-21-RK9P4P9L0 and tRF-16-PSQP4PE	tRF-5	LUAD tissues and cell lines	46
Breast cancer (BC)	tsRNA-26576	-	BC tissues	47
	ts-112	tRFs	BC cell lines	48
	tRF-Arg-CCT-017, tRF-Gly-CCC-001, and tiRNA-Phe-GAA-003	i-tRF and 5'tiRNA	plasmas	49
Triple-negative breast cancer (TNBC)	tDR-0009 and tDR-7336	-	Cell lines	50
Notriple-negative breast cancer (Non-TNBC)	tDR-7816	tRFs	Blood samples	51
Gastric cancer (GC)	tRF-33-P4R8YP9LON4VDP	-	peripheral blood samples	52
	tiRNA-Val-CAC-001	tRF-5	GC tissues and cells	53
	tRF-Val-CAC-016	-	GC tissues	54
	tRF-3019a	tRF-3	GC tissues and cells	55
	tRF-31-U5YKFN8DYDZDD	i-tRF	GC tumor tissues, sera, and cell lines	56
	tRF-5026a (tRF-18-79MP0P04)	tRFs	GC tissues and plasma samples	57
	tRF-19-3L7L73JD	tRFs	plasma and cell lines	58
	Has-tsr016141	tRFs	GC tissues and sera	59
Hepatocellular carcinoma (HCC)	ts-N102, ts-N59 and ts-N41	-	liver primary tumor tissues	60
	tRF-Gln-TTG-006	-	HCC sera	61
Liver cancer	tRNA-ValTAC-3, tRNA-GlyTCC-5, tRNA-ValAAC-5 and tRNA-GluCTC-5	-	plasma exosomes	62
Colorectal cancer (CRC)	tRF-22-WB86Q3P92, tRF-22-WE8SPOX52, tRF-22-WE8S68L52 and tRF-18-8R1546D2	-	Sequence Read Archives (SRA) public repository	63
	tiRNA-1:33-Pro-TGG-1 (5′tiRNA-Pro-TGG)	5'tiRNA	Sessile serrated lesions (SSLs) tissues	64
	5'-tiRNA-Val	5'tiRNA	CRC tumor tissues	65
	5'tiRNA-His-GTG	5'tiRNA	CRC tissues	66
Pancreatic ductal adenocarcinoma (PDAC)	tsRNA-ValTAC-41, tsRNA-MetCAT-37 and tsRNA-ThrTGT-23	tRF-3 and i-tRF	PDAC sera and tissue	67
Pancreatic cancer (PC)	tRF-Pro-AGG-004, and tRF-Leu-CAG-002	-	PC sera	68
Clear cell renal cell carcinoma (ccRCC)	5'-tRNA-Arg-CCT, 5'-tRNA-Glu-CTC and 5'-tRNA-Lys-TTT	5'tiRNA	cancerous tissues and sera	69
Prostate cancer (CaP)	5'-tRNA-Asp-GUC-half and 3'-tRNA-Asp-GUC-half	5'tiRNA and 3'tiRNA	prostate tissues	70
Muscle-invasive bladder cancer (MIBC)	tiRNA-1:33-Gly-GCC-1, tRF-1:32-Gly-GCC-1	5'tiRNA and tRF-5c	MIBC tissues	71
	tRF-+1:T20-Ser-TGA-1	tRF-1	MIBC tissues	71
Endometrial carcinoma (EC)	tRF-20-S998LO9D	tRF-5	EC tissues and serumal exosomes	72
High-grade serous ovarian cancer (HGSOC)	tRF-03357 and tRF-03358	tRFs	HGSOC sera and cells	73
Gliomas	tRFdb-3003a and tRFdb-3003b	tRF-3	tRF explorer and tRFdb databases	74
Glioblastomas (GBMs)	tRF-1-32-chrM.Lys-TTT, tiRNA-1-33-Gly-GCC-1, tiRNA-1-33-Gly-CCC-1, tRF-1-31-His-GTG-1, and tiRNA-1-33-Gly-GCC-2-M3	-	Fresh tumor tissues	75
	tiRNA-1-34-Lys-CTT-1-M2	-	Fresh tumor tissues	75
Diffuse glioma	ts-26, tRFdb-3012a, and tRFdb-3012b	tRF-1 and tRF-3	tRF explorer and tRFdb databases	76
Osteosarcoma	tRF-33-6SXMSL73VL4YDN, tRF-32-6SXMSL73VL4YK, tRF-32- M1M3WD8S746D2, tRF-35-RPM830MMUKLY5Z, tRF-33-K768WP9N1EWJDW and tRF-32-MIF91SS2P46I3	tRFs	Blood samples	77
Chronic lymphocytic leukemia (CLL)	ts-46 and ts-47	-	Blood samples	78
	ts-3676 and ts-4521	-	Blood samples	78
	ts-42, ts-70 and ts-36	-	Blood samples	79
	ts-43 and ts-44	tRF-5	Blood samples	79
Acute myeloid leukemia (AML)	tsRNA20 and tsRNA66	-	Blood samples	80
Nasopharyngeal carcinoma (NPC)	tRF-1:28-Val-CAC-2 and tRF-1:24-Ser-CGA-1-M3	tRF-5c and tRF-5b	NPC tissues	81
	tRF-55:76-Arg-ACG-1-M2	tRF-3b	NPC tissues	81
Oral submucous fibrosis (OSF)	tiRNA-Val-CAC-002	5'tiRNA	buccal OSF tissues	82
	tRF-Gly-TCC-016	tRF-5c	buccal OSF tissues	82
Papillary thyroid cancer (PTC)	tRF-39-0VL8K87SIRMM12E2	tRF-3	PTC tissues	83
Laryngeal squamous cell carcinoma (LSCC)	tRF-33-Q1Q89P9L842205	5'tiRNA	LSCC tumor tissues	84
Esophageal squamous cell carcinoma (ESCC)	tRNA-GlyGCC-5	-	Salivary exosomes	85

**Table 2:** tsRNAs for tumor early-stage screening and diagnosis

Tumour type	tsRNA	Expression	Screening or diagnosis	Refs
NSCLC	tRF-Leu-CAG	High	Diagnosis	44
LUAD	tRF-21-RK9P4P9L0 and tRF-16-PSQP4PE	High	Screening	46
BC	tsRNA-26576	High	Screening	47
	tRF-Arg-CCT-017, tRF-Gly-CCC-001 and tiRNA-Phe-GAA-003	High	Diagnosis	49
Non-TNBC	tDR-7816	Low	Screening	51
HCC	ts-N102	High	Diagnosis	60
	tRF-Gln-TTG-006	High	Diagnosis	61
Liver cancer	tRNA-ValTAC-3, tRNA-GlyTCC-5, tRNA-ValAAC-5 and tRNA-GluCTC-5	High	Screening	62
CRC	tRF-22-WB86Q3P92, tRF-22-WE8SPOX52, tRF- 22-WE8S68L52 and tRF-18-8R1546D2	Low	Diagnosis	63
PDAC	tsRNA-ValTAC-41 and tsRNA-MetCAT-37	High	Diagnosis	67
PC	tRF-Pro-AGG-004 and tRF-Leu-CAG-002	High	Diagnosis	68
ccRCC	5'-tRNA-Arg-CCT, 5'-tRNA-Glu-CTC, and 5'-tRNA-Lys-TTT	Low	Diagnosis	69
CaP	5'-tRNA-Glu-CUC-half	High	Diagnosis	70
EC	tRF-20-S998LO9D	Low	Screening	72
Diffuse glioma	ts-26, tRFdb-3012a, and tRFdb-3012b	Low	Screening	76
NPC	tRF-1:28-Val-CAC-2	High	Screening	81
OSF	tRF-Gly-TCC-016	High	Screening	82
PTC	tRF-39-0VL8K87SIRMM12E2	High	Diagnosis	83
LSCC	tRF-33-Q1Q89P9L842205	Low	Diagnosis	84
ESCC	tRNA-GlyGCC-5	High	Diagnosis	85

**Table 3:** tsRNAs used for tumor targeted therapy and application

Tumor type	tsRNA	Targeted gene	Mechanism	Therapeutic efficacy	Refs
NSCLC	tRF-Leu-CAG	AURKA	Decreases proliferation and cause G0/G1 cell-cycle progression via signaling pathways by targeting AURKA.	Well	44
GC	tiRNA-Val-CAC-001	LRP6	Decreases metastasis and proliferation and promote apoptosis via Wnt/β-catenin pathway by targeting LRP6.	High	53
	tRF-Val-CAC-016	CACNA1d	Suppress proliferation via MAPK pathway by targeting CACNA1d.	High	54
EC	tRF-20-S998LO9D	SESN2	Decreases migration, proliferation, and invasion and promote apoptosis by upregulating SESN2.	High	72
HGSOC	tRF-03357	HMBOX1	Decreases proliferation, migration, and invasion partly through downregulating HMBOX1.	Well	73
Gliomas	tRFdb-3003a/b	VAV2	Inhibit cell proliferation and tumor growth by directly binding to VAV2 to regulate its expression.	High	74
	tRFdb-3003a/b and ts-26	RBM43 and HOXA13	Play a tumor suppressor role in progression by directly targeting 3'untranslated region of RBM43 and HOXA13.	Medium	76
LSCC	tRF-33-Q1Q89P9L842205	PIK3CD	Induces apoptosis and inhibit cell growth, migration and invasion via directly downregulating PIK3CD.	High	84
CRC	5'tiRNA-His-GTG	LATS2	Decreases proliferation and anti-apoptosis via “turning off” hippo signaling pathway by targeting LATS2.	Well	66
TNBC	tDR-0009 (tDR-7336)	STAT3	Inhibits activation of STAT3 phosphorylation.	High	50
CLL	ts-46 and ts-47	-	-	Medium	78
	ts-42	-	Inactivates mostly by promoter methylation.	Medium	79
OSF	tiRNA-Val-CAC-002	-	Inhibits proliferation and invasion of cells and regulates MET.	Medium	82

**Table 4:** tsRNAs in the prognosis of tumors

Tumor type	tsRNA	Expression	Prognosis	Refs
LUAD	tsRNA-5001a	High	Poor	45
	tRF-21-RK9P4P9L0			46
BC	tRF-Arg-CCT- 017 and tiRNA-Phe-GAA-003	High	Poor	49
HCC	ts-N22	Low	Well	60
CRC	5'tiRNA-Pro-TGG	High	Poor	64
PDAC	tsRNA-ValTAC-41	High	Poor	67
PC	tRF-Pro-AGG-004 and tRF-Leu-CAG-002	High	Poor	68
Diffuse glioma	ts-26, tRFdb-3012a and tRFdb-3012b	Low	Well	76
Osteosarcoma	tRF-33-6SXMSL73VL4YDN, tRF-32-6SXMSL73VL4YK, tRF-32-M1M3WD8S746D2, tRF-35-RPM830MMUKLY5Z, tRF-33-K768WP9N1EWJDW, and tRF-32-MIF91SS2P46I3	High	Poor	77
CLL	ts-3676 and ts-4521	High	Poor	78
AML	tsRNA20 and tsRNA66	Low	Well	80

## Abbreviations

AML: Acute Myeloid Leukem-ia
ANG: Angiogenin
BC: Breast cancer
CaP: prostate cancer
ccRCC: clear cell renal cell carcinoma
CLL: Chronic lymphocytic leukemia
CRC: Colorectal cancer
EC: Endometrial carcinoma
ESCC: Esophageal squamous cell carcinoma
GC: Gastric cancer
GBMs: Glioblastomas
HCC: Hepatocellular arcinoma
HGSOC: High-grade serous ovarian cancer
LSCC: laryngeal squamous cell carcinoma
LUAD: Lung adenocarcinoma
miRNA: microRNA
MIBC: Muscle-invasive bladder cancer
NPC: Nasopharyngeal carcinoma
NSCLC: Non-small cell lung cancer
nts: nucleotides
OSF: Oral submucous fibrosis
PC: Pancreatic cancer
PDAC: Pancreatic ductal adenocarcinoma
piRNA: PIWI-interacting RNA
pre-tRNAs: precursor tRNAs
PTC: Papillary thyroid cancer
RNase Z/ELAC2: endonuclease Z
siRNA: small interfering RNA
sncRNAs: small non-coding RNAs
snRNA: small nuclear RNA
snoRNA: small nucleolar RNA
tiRNAs(tRHs): tRNA halves
TNBC: Triple-negative breast cancer
Non-TNBC: Notriple-negative breast cancer
tRFs: tRNA-derived fragments
tsRNA: tRNA-derived small RNA
